# Is staying overnight in a farming hut a risk factor for malaria infection in a setting with insecticide-treated bed nets in rural Laos?

**DOI:** 10.1186/1475-2875-9-372

**Published:** 2010-12-23

**Authors:** Daisuke Nonaka, Sakhone Laimanivong, Jun Kobayashi, Keobouphaphone Chindavonsa, Shigeyuki Kano, Viengxay Vanisaveth, Junko Yasuoka, Samlane Phompida, Masamine Jimba

**Affiliations:** 1 Department of Community and Global Health, Graduate School of Medicine, the University of Tokyo, 7-3-1 Hongo, Bunkyo, Tokyo, Japan; 2 Department of Epidemiology and International Health, International Clinical Research Center, National Center for Global Health and Medicine, 1-21-1 Toyama, Shinjuku, Tokyo, Japan; 3 Center of Malariology, Parasitology, and Entomology, Ministry of Health, Lao PDR, Vientiane, Lao PDR; 4 Department of International Medical Cooperation, National Center for Global Health and Medicine, 1-21-1 Toyama, Shinjuku, Tokyo, Japan; 5 Department of Tropical Medicine and Malaria, Research Institute, National Center for Global Health and Medicine, 1-21-1 Toyama, Shinjuku, Tokyo, Japan

## Abstract

**Background:**

Overnight stays in farming huts are known to pose a risk of malaria infection. However, studies reporting the risk were conducted in the settings of poor net coverage. This study sought to assess whether an overnight stay in a farming hut is associated with an increased risk of malaria infection if insecticide-treated bed nets (ITNs) are properly used.

**Methods:**

A pair of cross-sectional surveys was carried out in the Lamarm district of Sekong province, Laos, in March (dry season) and August (rainy season) in 2008. Questionnaire-based interviews and blood examinations were conducted with farmers and their household members from three randomly selected villages in March (127 households, 891 people) and August (128 households, 919 people). Logistic regression analysis, adjusted for potential confounding factors, was used to assess the association between malaria infection status and frequency of overnight stays for the two weeks prior to the study in both the seasons.

**Results:**

In March, 13.7% of participants reported staying overnight in a farming hut at least once in the previous two weeks. The percentage increased to 74.6% in August. Not only adults but also young children stayed overnight as often as adults. The use of an ITN the preceding night was common both in farming huts (66.3% in March, 95.2% in August), and in main residences (85.8% in March, 92.5% in August). Logistic regression analysis showed no statistical association between malaria infection status and frequency of overnight stays in farming huts in either study period. However, people sharing one family type net with five people or more were significantly more likely to have malaria than those sharing a net with up to two people in the dry season.

**Conclusions:**

This study showed that staying overnight in farming huts was not associated with an increased risk of malaria infection in the setting where ITNs were widely used in farming huts. It suggests that malaria infection during overnight stays in farming huts might be preventable if ITNs are properly used in rural Laos.

## Background

Laos is located in Southeast Asia, where malaria transmission is intensive in and on the fringes of forests [1]. With 70% of the population at risk, malaria is the leading cause of morbidity and mortality in the country [2]. Most of the population lives in rural areas and 84% of the households are engaged in rice farming [3]. The National Malaria Control Programme has emphasized the promotion of insecticide-treated bed nets (ITNs), early diagnosis, and prompt treatment as the principal malaria control strategies [4].

During the rice farming season, farmers and their family members often move from their village to a farm, where they stay in a temporary shelter (farming hut). Likewise, in Thailand, movements entailing overnight stays on farms occur throughout the year for rice cultivation, and peaked between June and September [5]. A study in Tanzania showed that most of the studied population stayed overnight in huts for weeding in February and harvesting rice in May [6].

These seasonal movements in relation to agricultural activities have been identified as risk factors associated with malaria infection [5,7-11]. In Thailand, the relative risk of infection for people who slept in farm huts was three times higher than that of people staying in residential villages [5]. In Vietnam, farmers who regularly slept in plot huts in the forest were approximately three times more likely to be infected with malaria than those involved in other work [7].

Two environmental factors for higher risk of malaria infection have been identified among those who stayed overnight in farm huts. First, malaria transmission is more intense in the farm huts than in the village settlements. Second, farm huts are poorly constructed with fewer barriers against mosquitoes [5]. In addition to these environmental factors, social factors were also noted. A shortage of nets leads people to keep their valued nets in their village houses, which are more secure and it may be cumbersome for people to carry and set up their nets in their farm huts [12].

Recently, the Global Fund to Fight AIDS, Tuberculosis and Malaria and other global efforts rapidly increased the coverage of ITNs in many malaria endemic countries. In Laos, ITN coverage reached 77.5% of the target population in 2003 [13]. Furthermore, 1,672,500 ITNs, including long-lasting ITNs, were distributed amongst the population of five million between 2004 and 2007 [14]. With the assumption that two people use a net together, this covered 67% of the total population.

Although the coverage has been increased in many countries, few studies have documented the association between malaria infection risk and overnight stay in a farming hut in a setting of high ITN coverage. Most of the previous studies about overnight stay provided little information on whether ITNs or any nets were used among their target populations [5,7-9], and others were conducted in the settings where no use or use of untreated bed nets was more common than use of ITNs [10,11]. Thus, this study sought to assess whether staying overnight in a farming hut is associated with malaria infection among farmers and their family members in a rural district of Laos.

## Methods

### Study site

This study was carried out in the Lamarm district of Sekong province, in the southern region of Laos in 2008. The annual incidence of malaria in 2003 was 15.4 cases per 1,000 people in the province, the highest in the country [2]. Falciparum malaria accounted for more than 95% of the confirmed cases [13]. The rainy season is from April to November, with peak rainfall in July or August [15]. As irrigation is not well developed in most areas, the rice farming season coincides with the rainy season.

Sekong province consists of four districts. With 27,000 people and 66 villages, Lamarm is the most populous district. This district was selected because access to villages in rainy seasons is known to be very difficult in other two districts. The remaining one district contained only a small number of villages. Governmental healthcare facilities in the Lamarm district include one provincial hospital and four health centres that serve rural communities.

In the district, the National Malaria Control Programme has been annually distributing conventional bed nets since 2000. During distribution, bed nets are sold in communities at a subsidized price (8,000 Kip: approximately 1 USD); insecticide treatment services are provided for free. Since 2009 long-lasting ITNs have been distributed instead of conventional nets with insecticide treatment. Indoor residual spraying has not been adopted. The use of personal spray, mosquito coil, and repellent appeared to be rare.

For the target village selection, two-stage cluster random sampling was used. First, one out of the four health centres was randomly selected, and then three of the ten villages were randomly selected from the catchment area of the health centre.

### Study population

From the three selected villages (Village A, B, and C), a total of 143 households (85 in Village A, 41 in Village B, and 17 in Village C) were registered in the village census. An attempt was made to include all of the households in a survey. Data were collected from 134 households in the March survey, and 135 in the August survey. In both the surveys, seven households that were not engaged in farming were excluded. In total, data of 127 households (72 in Village A, 39 in Village B, and 16 in Village C) with 891 people (445 in Village A, 262 in Village B, and 184 in Village C) and 128 households (76 in Village A, 37 in Village B, and 15 in Village C) with 919 people (472 in Village A, 253 in Village B, and 194 in Village C) were analysed in the March survey and in the August survey, respectively. Of the 127 households surveyed in March, 104 with 761 people were included in both surveys.

### Variables and measurements

The outcome in this study was the infection status of *Plasmodium falciparum*, as measured by rapid diagnosis test (Paracheck Pf^®^, Orchid Biomedical Laboratories, Goa). Malaria infection was defined by positive test result with or without clinical symptoms. The independent variable was the frequency of overnight stays in farming huts in the two weeks prior to each survey, as measured by a question with possible response options such as 0 day, 1-4 day, and 5 days or more. Because the respondents of an interview pre-test had difficulty in answering the number of exact days where they slept in huts, the question was asked in the categorical manner. Other variables included socio-demographic variables (sex, age, and number of household members), socio-economic variables (educational attainment of household head and household possession of assets, such as radio, bicycle, motorbike, and car), bed net-related variables (use of ITN, number of people sharing the same family type net (with or without insecticide treatment), presence of hung ITN in main residence, and presence of hung ITN in hut), rice farming-related variables (type of rice farming and distance to farming hut), and a cluster-related variable (village). These variables were measured by conducting interviews with household members and observing the bed nets.

In this study, ITN was defined as a conventional net, which was treated with insecticide in the past twelve months. The possibility that households possessed a long-lasting ITN was not taken into account, not only because the National Malaria Control Programme had not provided long-lasting ITNs in the study site, but also because they were rarely available at retailers. The use of ITN was defined as the sleeping under an ITN during the previous night.

### Reliability and validity of the measurements

Paracheck Pf^® ^is a rapid diagnostic test kit which detects *Plasmodium falciparum*-specific protein (histidine-rich protein-2) in human blood. In Laos, this test tools are widely used in two settings: one is at rural health centres where microscopy examinations are often unavailable and another is by village health volunteers in communities [16]. A review study reported that histidine-rich protein-2-based tests including Paracheck commonly give sensitivity > 90% and specificity >85% in clinical cases [17]. Paracheck test results performed by village health volunteers in Laos showed that sensitivity, specificity, positive predictive value, and negative predictive value were 74.3%, 99.3%, 83.9%, and 98.7%, respectively [16].

With the exception of four variables (frequency of overnight stays in farming huts, number of people sharing the same family type net, distance to farming hut, and rice farming type), measurements were based on questions drawn from the Multiple Indicator Cluster Survey Questionnaire 3 developed by the United Nations Children's Fund. Surveyors also confirmed the presence and type of bed nets left in each household's main residence. To increase the validity of the measurements, interview procedures were pre-tested with surveyors at 25 households in a village. Questions that respondents had difficulty in answering were modified.

### Data collection

A pair of cross-sectional surveys was conducted in March (during the dry season) and August (during the rainy season), 2008. Each survey consisted of interviews with household members with particular focus on heads and blood examinations aiming at all household members.

Five health workers from local health offices were recruited and trained as surveyors. They made household visits and conducted interviews with household heads. The surveyors invited a household head to be the main interviewee. In cases where the head could not participate in the interview, the next responsible person (e.g. a spouse) was chosen. Other household members were encouraged to help the household head to correctly respond to the interview questions.

Blood examinations of villagers to determine the prevalence of *P. falciparum *in the villages were conducted on the same days as the interviews. All villagers were invited to assemble at a mobile laboratory that was set up at a designated place including a village health volunteer's house and a primary school. Blood samples were collected from those who gathered and consented to their participation. For child participants, their guardians consented to their participation. Two laboratory technicians from the Center of Malariology, Parasitology, and Entomology, collected a finger prick blood sample from the participants for testing by rapid diagnosis test. Collected blood was immediately used for testing. Results of Paracheck assay were read at 15 minutes from the starting of testing and interpreted by both the technicians. In case that Paracheck turned out to be invalid or technicians did not reach an agreement on interpretation, a blood sample was collected for re-testing. Laotian medical doctors from the same institute treated all participants who tested positive by giving Coartem^® ^or artesunate suppository according to the National Malaria Treatment Guideline. Except one field surveyor, the same members worked for the data collection both in the March and August surveys.

### Statistical analysis

A sample size of 516 people was needed to detect a statistically significant difference in malaria prevalence between people who stay overnight in farming huts and those do not, with 80% power at 95% significance level. It was hypothesized that 90% of villagers would be farmers or a farmer's family members and that half of them would have stayed overnight in a farming hut in the two weeks prior to the survey. It was also hypothesized that 70% of them would participate in a blood examination on the basis of the study conducted in the district [18], and the difference in the prevalence of malaria would be 10% among people who would stay overnight in a farming hut (estimated at 20%) and those who would not (10%). The sample size was increased to 700 people to protect against uncertainties in the sample size estimations.

Bivariate and multivariate analyses were done to examine the association between the outcome variable and both independent and confounding variables. For the analysis, each confounding variable was categorized as follows: age (< 5 years, 5-14 years, or ≥15 years); number of household members (< 4 people, 4-9 people, or ≥10 people); educational attainment (no education, primary, or secondary and above); possession of asset (no radio or bicycle/motorbike/car, radio or bicycle without motorbike/car, or motorbike/car); number of people sharing the same family type net (1-2 people, 3-4 people, or ≥5 people); distance to farming hut (< 3 km or ≥3 km); and type of rice farming (paddy only, slash-and-burn only, both). Odds ratios (OR) and 95% confidence intervals (CI) of the outcome for each variable were estimated using logistic regression analysis. The multivariate model was adjusted for all the variables other than the independent variable (frequency of overnight stays). Statistical analysis was performed using SPSS 17.0 (SPSS Inc., Chicago, IL). A *P*-value of < 0.05 was accepted as statistically significant.

### Ethical clearance

This study was approved by the National Ethics Committee for Health Research, Ministry of Health, Lao People's Democratic Republic (No. 044/NECHR). The participants knew that their participation would be voluntary and that all data obtained would be confidential. Verbal and written consent was obtained from all the participants before conducting the survey.

## Results

### Household characteristics

The mean number of household members was 7.0 (891/127) in the March survey, and 7.2 (919/128) in the August survey (Table 1). Educational attainment of the household heads was mostly primary level. Most of the households (74.8% in March, 71.9% in August) practiced paddy rice farming with or without slash-and-burn rice farming and the remainder exclusively practiced slash-and-burn rice farming. In both surveys, the median distance between the permanent residence and the rice paddies or slash-and-burn field was 3.0 km. Farming huts were constructed of wood and/or bamboo with a thatched roof. The front sides were largely or completely open. Although most main residences had a window without mosquito-proof net, main residences appeared to be more protected than farming huts in terms of mosquito invasions.

**Table 1:** Household characteristics.

		March (n = 127)			August (n = 128)		
			
		n	%	**95% CI**^**a**^	n	%	**95% CI**^**a**^
Mean number of household members (SD)		7.0	(4.1)	6.3, 7.8	7.2	(4.3)	6.4, 7.9
House type							
	Bamboo	36	28.3	13.6, 43.0	28	21.9	6.6, 37.2
	Wood	90	70.9	61.5, 80.3	99	77.3	69.0, 85.6
	Cement	1	0.8	-16.7, 18.3	1	0.8	-16.7, 18.3
Educational attainment of household head							
	No	21	16.5	0.6, 32.4	17	13.3	-2.8, 29.4
	Primary	85	66.9	56.9, 76.9	90	70.3	60.9, 79.7
	Secondary or above	21	16.5	0.6, 32.4	21	16.4	0.6, 32.2
Farm type							
	Paddy	47	37.0	23.2, 50.8	56	43.8	30.8, 56.8
	Slash	32	25.2	10.2, 40.2	36	28.1	13.4, 42.8
	Paddy and slash	48	37.8	24.1, 51.5	36	28.1	13.4, 42.8
Household assets							
	None	45	35.4	21.4, 49.4	49	38.3	24.6, 51.8
	Radio and/or bicycle	48	37.8	24.1, 51.5	44	34.4	20.4, 48.4
	Motorbike and/or car	34	26.8	11.9, 41.7	35	27.3	12.5, 42.1

^a^: 95% confidence interval for the estimated proportion or mean

### Individual characteristics

The total population reported from the households was 891 in the March survey and 919 in the August survey (Table 2). Adults (15 years old or older) and females accounted for nearly a half of the population.

**Table 2:** Individual characteristics.

		March (n = 891)			August (n = 919)		
			
		n	%	95% CI^a^	n	%	95% CI^a^
Age (years)							
	<5	159	17.8	11.9, 23.7	172	18.7	12.9, 24.5
	5-14	273	30.6	25.1, 36.1	277	30.1	24.7, 35.5
	≥15	459	51.5	46.9, 56.1	470	51.1	46.6, 55.6
Sex							
	Male	433	48.6	43.9, 53.3	446	48.5	43.9, 53.1
	Female	458	51.4	46.8, 56.0	473	51.5	47.0, 56.0
Frequency of sleeping in hut (number of days during past two weeks)							
	0	769	86.3	83.9, 88.7	234	25.5	19.9, 31.1
	1-4	58	6.5	0.2, 12.8	223	24.3	18.7, 29.9
	≥5	64	7.2	0.9, 13.5	462	50.3	45.7, 54.9
Slept under net (any type) the preceding night							
	Yes	870	97.6	96.6, 98.6	916	99.7	99.3, 100.1
	No	6	0.7	-6.0, 7.4	3	0.3	-5.9, 6.5
	Unknown	15	1.7	-4.8, 8.2	0	0.0	
Slept under insecticide-treated net the preceding night (at hut)							
	Yes	53	66.3	53.6, 79.0	555	95.2	93.4, 97.0
	No	3	3.8	-17.8, 25.4	9	1.5	-6.4, 9.4
	Unknown	24	30.0	11.7, 48.3	19	3.3	-4.7, 11.3
Slept under insecticide-treated net the preceding night (at main residence)							
	Yes	680	85.8	83.2, 88.4	309	92.5	89.6, 95.6
	No	48	6.1	-0.7, 12.9	6	1.8	-8.8, 12.4
	Unknown	65	8.2	1.5, 14.9	19	5.7	-4.7, 16.1
Slept under insecticide-treated net the preceding night (overall)							
	Yes	733	82.3	79.5, 85.1	864	94.0	92.4, 95.6
	No	55	6.2	-0.2, 12.6	15	1.6	-4.8, 7.9
	Unknown	103	11.6	5.4, 17.8	40	4.3	-2.0, 10.6
Number of people sharing any one net							
	1-2	276	31.0	25.5, 36.5	375	40.8	35.8, 45.8
	3-4	435	48.8	44.1, 53.5	420	45.7	40.9, 50.5
	≥5	159	17.8	11.9, 23.7	121	13.2	7.2, 19.2
	Not using a net/unknown	21	2.4	-4.1, 8.9	3	0.3	-5.9, 6.5

^a^: 95% confidence interval for the estimated proportion

In the March survey, 13.7% of the population reported that they stayed overnight in a farming hut in the two weeks prior to the survey. In contrast, 74.6% reported doing so in the August survey. Children under five years who stayed overnight in farming huts accounted for 20.8% (33/159) in the March survey and 80.2% (138/172) in the August survey. These percentages were higher than any other groups: for children aged 5-14 years the percentages were 7.7% (21/273) in the March survey and 75.1% (208/277) in the August survey; for adults there were 14.8% (68/459) in the March survey and 72.1% (339/470) in the August survey.

Use of ITN was common both in farming huts and main residence. In the March survey, 66.3% (53/80) of those who stayed overnight in farming huts used an ITN, and 85.8% (680/793) of those who stayed overnight in their main residence used one. In the August survey, 95.2% (555/583) of those who stayed overnight in farming huts used an ITN, and 92.5% (309/334) who stayed overnight in their main residence used one.

People commonly shared one net with multiple people. In the March survey, the most commonly reported number of people sharing a net was three to four (48.8%; 435/891), followed by one to two (31.0%; 276/891) and five or more (17.8%; 159/891). Likewise, in the August survey the most commonly reported number of people sharing was three to four (45.7%; 420/919), followed by one to two (40.8%; 375/919) and five or more (13.2%; 121/919).

For blood examination, 73.8% (658/891) and 69.4% (638/919) participated in the March and August surveys, respectively. The prevalence of falciparum malaria was 15.3% (101/658) in the March survey and 22.9% (146/638) in the August survey. Of the people who participated in the blood examination in March, 64 (positive: 16, negative: 48) had self-reported fever that occurred in the past one week and the remaining 594 (positive: 85, negative: 509) had no fever. Of the people who participated in the blood examination in August, 60 (positive: 26, negative: 34) had fever and the remaining 578 (positive: 120, negative: 458) had no fever. Of the 101 people who were tested positive in March, 18 were also tested positive, 59 negative, and 24 not participating in August.

### Bed net characteristics

In total, there were 528 nets in the March survey and 560 in the August survey (Table 3). In the March survey, 61.9% were used in the main residence, while 32.9% were not used either in the main residence or farming hut, although some of them were hung. In the August survey, 43.2% and 29.1% were used in the farming hut and main residence, respectively. The remainder (27.7%) was not used either in the main residence or farming hut. Insecticide-treated nets accounted for 83.5% of the total nets in the March survey and 93.6% in the August survey. Almost all were family type nets, which can cover two to three people. The mean number of nets per household was 4.2 (528/127) in the March survey, and 4.4 in the August survey.

**Table 3:** Household bed net characteristics.

		March (n = 528)			August (n = 560)		
			
		n	%	95% CI^a^	n	%	95% CI^a^
Place where any nets were used/not used the night before the survey							
	Placed in main residence and used	327	61.9	56.6, 67.2	163	29.1	22.1, 36.1
	Placed in main residence but not used^b^	65	12.3	4.3, 20.3	106	18.9	11.4, 26.4
	Placed in farming hut and used	27	5.1	-3.2, 13.4	242	43.2	37.0, 49.4
	Placed in farming hut but not used^b^	109	20.6	13.0, 28.2	49	8.8	0.9, 16.7
Net type							
	Family	525	99.4	98.7, 100.0	557	99.5	98.9, 100.1
	Double	2	0.4	-8.3, 9.1	2	0.4	-8.3, 9.1
	Single	1	0.2	-8.6, 9.0	1	0.2	-8.6, 9.0
Insecticide treatment status							
	Treated	441	83.5	80.0, 87.0	524	93.6	91.5, 95.7
	Not treated	35	6.6	-1.6, 14.8	12	2.1	-6.0, 10.2
	Unknown	52	9.8	1.7, 17.9	24	4.3	-3.8, 12.4
Mean number of any nets per household (SD)							
	Main residence	3.1	(1.6)	2.8, 3.4	2.1	(1.6)	1.8, 2.4
	Hut	1.1	(1.3)	0.9, 1.3	2.3	(1.7)	2.0, 2.6
	Overall	4.2	(2.1)	3.8, 4.5	4.4	(1.9)	4.0, 4.7
Mean number of ITNs per household (SD)							
	Main residence	2.7	(1.7)	2.4, 3.0	2.0	(1.7)	1.7, 2.3
	Hut	0.9	(1.2)	0.7, 1.1	2.1	(1.7)	1.8, 2.4
	Overall	3.5	(2.3)	3.1, 3.9	4.1	(2.1)	3.7, 4.5

^a^: 95% confidence interval for the estimated proportion or mean

^b^: These unused nets included both hung and not hung nets.

### Factors associated with malaria infection status

Logistic regression analysis for the March survey showed no association between malaria infection status and frequency of overnight stays in huts even after adjustment for the confounding variables (Additional File 1). Among variables other than frequency of sleeping in huts, "number of people sharing the same family type net with or without insecticide treatment" and "rice farming type" were associated with increased risk of malaria infection in the multivariate model. People who shared one net with five people or more had a greater risk of malaria infection than those sharing one net with up to two people (adjusted OR 2.22, 95% CI 1.15-4.27). Additionally, people who predominately practiced slash-and-burn farming were more likely to be infected with malaria than those practicing paddy farming alone (adjusted OR 2.12, 95% CI 1.03-4.35).

In the August survey, logistic regression analysis showed no association between malaria infection status and frequency of overnight stays in huts even after adjustment for potential confounding variables (Additional File 2). Among the confounding variables, "rice farming type" was found to be associated with the risk of malaria infection in the bivariate model. After adjustment, however, the variable was found to be not statistically significant. No statistical association was found with any other variables.

## Discussion

The primary finding was that overnight stays in farming huts was not associated with the risk of malaria infection in either the dry or rainy seasons in the study site in Laos, where ITNs were widely used both in permanent residences and farming huts.

This finding suggests that previously reported associations might be due partly to insufficient coverage or improper use of ITNs in the farming huts. This finding was in line with that of the Tanzania study that reported no association between overnight stays in the farming huts and incidence of febrile illness episodes in the malaria endemic area of the country [6]. In that study, 97.9% of the people who stayed overnight in farming huts reported that they used a bed net and nearly 60% of the nets were ITNs.

The finding is important not only because overnight stays in farming huts are reported in malaria endemic countries other than Laos, but because overnight stays in farming huts are not confined to adult population. As shown both in the present study and by Hetzel *et al *[6], children under five years of age, those most vulnerable to malaria infection, were taken to the farming huts by their parents. Although the coverage of ITNs is high in Laos, it remains low in many malaria endemic countries [13]. Efforts should be made to increase ITN coverage to protect this vulnerable group.

The second important finding was that using one family type net with five or more people significantly increased the odds of malaria infection risk. In the study site, almost all the nets were family type, which are usually designed to cover up to three adult people. Thus, sharing one family type net with five or more people quite obviously exceeds its capacity. The finding is in line with a study from Vietnam that reported that people sharing one net with four to six people were more likely to have malaria than those who shared with up to three people [19]. However, their results were not adjusted for potential confounding variables. Results of the present study showed that the association was found even after adjustment for age, the number of household members, and insecticide-treatment status.

The importance of health education regarding the proper use of nets has been recognized in malaria control [20,21]. However, such education does not necessarily cover how many people can share a net [22,23]. Additionally, Roll Back Malaria indicators, which are often used to assess progresses of malaria control efforts, focus only on whether people sleep under a net or not and pay little attention to proper net use [24]. Thus, this study suggests that number of people sharing one net should be emphasized in health education and be included as an indicator to assess the proper use of nets.

An interesting finding was that although the association between the number of people sharing the same family type net and malaria infection was statistically significant in the March survey, no association was found in the August survey. This difference might be related to the decreased repellent effects of ITNs. In the study site, the National Malaria Control Programme annually provides net treatment around April, and people have little chance to treat their nets with insecticide except the programme-led treatment exercise. Although the repellent effect on mosquitoes is strongest soon after treatment, the effect gradually decreases as time passes [25]. Thus, the reason for the lack of association in the August survey might be due to the effects from insecticide remaining on the nets. Alternatively, the finding could be due to the results of increased number of ITNs and its use. In the August survey, ITNs accounted for 93.6% of the total nets, while 83.5% in the March survey.

The results suggest that people who practice seasonal movements may need a greater number of nets than those who do not. In March, 25.7% of the total bed nets were placed in the farming huts even though the study respondents reported that they rarely stayed at the huts overnight. There were two possible reasons for this: first, the nets may be used for nuisance protection during the day, as was reported in a number of studies [26,27]; second, the difficulty involved with frequently carrying a net between the main residences and farming huts [12]. Programme planners need to take the extra nets used in farming huts into account when they decide how many nets are required for distribution.

In this study, blood examinations were performed with Paracheck, which is based on the detection of histidine-rich protein-2 produced by falciparum parasites. Because this protein can remain in blood for several weeks even after successful treatment, some of the study participants who tested positive might not have had parasitaemia (false-positive). However, it is unlikely that many of them resulted from false-positives; studies that assessed the performance of Paracheck among asymptomatic population in Asian settings showed that sensitivity, specificity, positive predictive value, and negative predictive value were 92.3%, 97.2%, 83.7%, and 98.8% in Thailand (prevalence: 15.0%), and 94.4%, 89.0%, 70.3%, and 98.3% in India (prevalence: 24.4%), respectively [28,29].

This study asked people's behaviors regarding overnight stay in farming huts in the past two weeks. The use of the two weeks duration is reasonable to minimize the potential recall bias associated with self-report, but may not be enough to assess an association between sleeping in farming hut and malaria infection, particularly when the extended period of persistence of histidine-rich protein-2 is considered. However, it is likely that the study population who frequently slept in farming huts for the past two weeks were more likely to choose to sleep in farming huts around the survey period, compared to those who never slept in farming huts.

In this study there were five major limitations. First, nearly 30% of the people did not participate in blood examinations. Logistic regression analysis was conducted using data from those who participated. Thus, results are not free from selection bias. Those who did not participate in blood examinations were more likely to sleep in huts for five days or more compared to those who participated in March (10.7% vs. 5.9%) and in August (52.1% vs. 48.7%). It suggests that one reason for non-participation might be due to people working in the farms and that the association between malaria infection and frequency of overnight stays might be underestimated. In a future study, performing blood examinations at the time of interview is recommended to avoid potential loss due to non-response. Second, because any entomological surveys were not conducted, it is unclear how intensive transmissions were in permanent residences and farming huts. Third, because the data collection was limited to only one health zone in the district, the samples of this study might not reflect the entire population of the district. However, as other health zones have the similar ecological setting and the same ethnic group (Lao Thueng), differences in malaria epidemiology and people's behaviors seem to be small. Fourth, the association might be somewhat underestimated in case sleeping in farming huts was associated with low-level parasitaemia. Finally, although the estimated minimum sample size was multiplied by 1.5, the increase might be insufficient to fully address the design effect of the sampling strategies used in this study. The estimated sample size should have been multiplied with consideration to design effect related to cluster surveys.

## Conclusions

This study showed that staying overnight in farming huts was not associated with an increased risk of malaria infection in the setting where ITNs were widely used in farming huts. It suggests that malaria infection during overnight stays in farming huts might be preventable if ITNs are properly used in rural Laos.

## Competing interests

The authors declare that they have no competing interests.

## Authors' contributions

DN was the principal investigator and was responsible for the entire process. SL, JK, KB, VH, and SP contributed to the development of the study design and coordinated the field work. JK, SK, JY, and MJ contributed to data analysis and to review of the manuscript. All authors read and approved the final manuscript.

## Supplementary Material

Additional file 1 **Logistic regression analysis which examined the associations of variables with malaria infection status in March survey**. Click here for file

Additional file 2 **Logistic regression analysis which examined the associations of variables with malaria infection status in August survey**. Click here for file

## References

[B1] Delacollette C D'Souza C Christophel E Thimasarn K Abdur R Bell D Dai TC Gopinath D Lu S Mendoza R Ortega L Rastogi R Tantinimitkul C Ehrenberg J Malaria trends and challenges in the Greater Mekong Subregion Southeast Asian J Trop Med Public Health 2009 40 674 691 19842400

[B2] WHO, UNICEF The World Malaria Report 2005 2005 Geneva: World Health organization

[B3] Rigg JD Forests, marketization, livelihoods and the poor in the Lao PDR Land Degrad Develop 2006 17 123 133 10.1002/ldr.719

[B4] Center of Malariology, Parasitology, and Entomology Country strategic plan to roll back malaria from 2001-2005 2000 Vientiane: Ministry of Health, Lao People's Democratic Republic

[B5] Somboon P Aramrattana A Lines J Webber R Entomological and epidemiological investigations of malaria transmission in relation to population movements in forest areas of north-west Thailand Southeast Asian J Trop Med Public Health 1998 29 3 9 9740259

[B6] Hetzel MW Alba S Fankhauser M Mayumana I Lengeler C Obrist B Nathan R Makemba AM Mshana C Schulze A Mshinda H Malaria risk and access to prevention and treatment in the paddies of the Kilombero Valley, Tanzania Malar J 2008 7 7 10.1186/1475-2875-7-7 18184430 PMC2254425

[B7] Erhart A Ngo DT Phan VK Ta TT Van Overmeir C Speybroeck N Obsomer V Le XH Le KT Coosemans M D'alessandro U Epidemiology of forest malaria in central Vietnam: a large scale cross-sectional survey Malar J 2005 4 58 10.1186/1475-2875-4-58 16336671 PMC1325238

[B8] Singhanetra-Renard A Malaria and mobility in Thailand Soc Sci Med 1993 37 1147 1154 10.1016/0277-9536(93)90254-2 8235754

[B9] Sevilla-Casas E Human mobility and malaria risk in the Naya river basin of Colombia Soc Sci Med 1993 37 1155 67 10.1016/0277-9536(93)90255-3 8235755

[B10] Matthys B Vounatsou P Raso G Tschannen AB Becket EG Gosoniu L Cissé G Tanner M N'goran EK Utzinger J Urban farming and malaria risk factors in a medium-sized town in Cote d'Ivoire Am J Trop Med Hyg 2006 75 1223 1231 17172397

[B11] Vythilingam I Sidavong B Chan ST Phonemixay T Vanisaveth V Sisoulad P Phetsouvanh R Hakim SL Phompida S Epidemiology of malaria in Attapeu Province, Lao PDR in relation to entomological parameters Trans R Soc Trop Med Hyg 2005 99 833 839 10.1016/j.trstmh.2005.06.012 16112154

[B12] Factors that affect the success and failure of insecticide treated net programs for malaria control in SE Asia and the Western Pacific http://www.who.int/malaria/publications/atoz/itn_r62.pdf

[B13] WHO, UNICEF The World Malaria Report 2008 2008 Geneva: World Health organization

[B14] Rattanaxay P Phompida S Kobayashi J Tongol-Rivera P, Kano S A review of malaria situation and its control in Lao PDR Asian Parasitology. Malaria in Asia 2005 6 Chiba, Japan; The Federation of Asian Parasitologists 85 104

[B15] Vythilingam I Phetsouvanh R Keokenchanh K Yengmala V Vanisaveth V Phompida S Hakim SL The prevalence of Anopheles (Diptera: Culicidae) mosquitoes in Sekong Province, Lao PDR in relation to malaria transmission Trop Med Int Health 2003 8 525 535 10.1046/j.1365-3156.2003.01052.x 12791058

[B16] Phommanivong V Thongkham K Deyer G Rene JP Barennes H An assessment of early diagnosis and treatment of malaria by village health volunteers in the Lao PDR Malar J 2010 9 347 10.1186/1475-2875-9-347 21122128 PMC3014971

[B17] Wongsrichanalai C Barcus MJ Muth S Sutamihardja A Wernsdorfer WH A review of malaria diagnostic tools: microscopy and rapid diagnostic test (RDT) Am J Trop Med Hyg 2007 77 6 Suppl 119 127 18165483

[B18] Nonaka D Vongseththa K Kobayashi J Bounyadeth S Kano S Phompida S Jimba M Public and private sector treatment of malaria in Lao PDR Acta Trop 2009 112 283 287 10.1016/j.actatropica.2009.08.013 19683502

[B19] Abe T Honda S Nakazawa S Tuong TD Thieu NQ Hung le X Thuan le K Moji K Takagi M Yamamoto T Risk factors for malaria infection among ethnic minorities in Binh Phuoc, Vietnam Southeast Asian J Trop Med Public Health 2009 40 18 29 19323029

[B20] Tanner M Vlassoff C Treatment-seeking behaviour for malaria: a typology based on endemicity and gender Soc Sci Med 1998 46 523 532 10.1016/S0277-9536(97)00195-0 9460831

[B21] Ibidapo CA Perception of causes of malaria and treatment-seeking behaviour of nursing mothers in a rural community Aust J Rural Health 2005 13 214 218 10.1111/j.1440-1584.2005.00704.x 16048462

[B22] Nonaka D Kobayashi J Jimba M Vilaysouk B Tsukamoto K Kano S Phommasack B Singhasivanon P Waikagul J Tateno S Takeuchi T Malaria education from school to community in Oudomxay province, Lao PDR Parasitol Int 2008 57 76 82 10.1016/j.parint.2007.09.005 17980652

[B23] Ayi I Nonaka D Adjovu JK Hanafusa S Jimba M Bosompem KM Mizoue T Takeuchi T Boakye DA Kobayashi J School-based participatory health education for malaria control in Ghana: engaging children as health messengers Malar J 2010 9 98 10.1186/1475-2875-9-98 20398416 PMC2865503

[B24] WHO/CDS/RBM Roll Back Malaria in the African Region: Framework for Monitoring Progress and Evaluating Outcomes and Impact 2000 25 WHO/CDS/RBM/2000

[B25] Lines J Review: mosquito nets and insecticides for net treatment: a discussion of existing and potential distribution systems in Africa Trop Med Int Health 1996 1 616 632 10.1111/j.1365-3156.1996.tb00087.x 8911446

[B26] Adongo PB Kirkwood B Kendall C How local community knowledge about malaria affects insecticide-treated net use in northern Ghana Trop Med Int Health 2005 10 366 378 10.1111/j.1365-3156.2005.01361.x 15807801

[B27] Yohannes K Dulhunty JM Kourleoutov C Manuopangai VT Polyn MK Parks WJ Williams GM Bryan JH Malaria control in central Malaita, Solomon Islands. 1. The use of insecticide-impregnated bed nets Acta Trop 2000 75 173 183 10.1016/S0001-706X(00)00055-3 10708657

[B28] Proux S Hkirijareon L Ngamngonkiri C McConnell S Nosten F Paracheck-Pf: a new, inexpensive and reliable rapid test for P. falciparum malaria Trop Med Int Health 2001 6 99 101 10.1046/j.1365-3156.2001.00694.x 11251904

[B29] Singh N Saxena A Sharma VP Usefulness of an inexpensive, Paracheck test in detecting asymptomatic infectious reservoir of plasmodium falciparum during dry season in an inaccessible terrain in central India J Infect 2002 45 165 168 10.1016/S0163-4453(02)91055-8 12387772

