# Supplementary material for: Is staying overnight in a farming hut a risk factor for malaria infection in a setting with insecticide-treated bed nets in rural Laos?
Source: Malar J. 2010 Dec 23;9:372. doi: 10.1186/1475-2875-9-372 (PMC3224235; doi:10.1186/1475-2875-9-372)
Supplement: Additional file 1 — Logistic regression analysis which examined the associations of variables with malaria infection status in March survey. [file 1475-2875-9-372-S1.DOC]

## Additional file 1

## Logistic regression analysis which examined the associations of variables with malaria infection status in March survey.

|  | Total | Positive  (Prevalence) | Bivariate | |  | Multivariate | |
| --- | --- | --- | --- | --- | --- | --- | --- |
|  | n=658 | n=101 (%) | OR^a^ | 95% CI^b^ |  | OR^a^ | 95% CI^b^ |
| Age (years) |  |  |  |  |  |  |  |
| <5 | 117 | 16 (13.7) | 1.00 |  |  | 1.00 |  |
| 5-14 | 240 | 41 (17.1) | 1.30 | 0.70-2.43 |  | 1.57 | 0.80-3.06 |
| >15 | 301 | 44 (14.6) | 1.08 | 0.58-2.00 |  | 1.34 | 0.69-2.61 |
| Sex |  |  |  |  |  |  |  |
| Female | 357 | 55 (15.4) | 1.00 |  |  | 1.00 |  |
| Male | 301 | 46 (15.3) | 0.99 | 0.65-1.52 |  | 0.98 | 0.63-1.52 |
| Number of household members |  |  |  |  |  |  |  |
| <5 people | 62 | 13 (21.0) | 1.00 |  |  | 1.00 |  |
| 5-9 people | 316 | 53 (16.6) | 0.75 | 0.38-1.48 |  | 0.59 | 0.28-1.25 |
| >10 people | 277 | 35 (12.6) | 0.55 | 0.27-1.11 |  | 0.55 | 0.24-1.28 |
| Household assets |  |  |  |  |  |  |  |
| No | 220 | 34 (15.5) | 1.00 |  |  | 1.00 |  |
| Radio and/or bicycle | 255 | 32 (12.5) | 0.79 | 0.47-1.32 |  | 0.91 | 0.50-1.66 |
| Motorbike and/or car | 183 | 35 (19.1) | 1.29 | 0.77-2.17 |  | 1.58 | 0.82-3.05 |
| Frequency of overnight stays in hut in the two weeks prior to the survey |  |  |  |  |  |  |  |
| 0 day | 561 | 85 (15.2) | 1.00 |  |  | 1.00 |  |
| 1-4 days | 48 | 6 (12.5) | 0.80 | 0.33-1.94 |  | 0.81 | 0.32-2.09 |
| >5 days | 39 | 7 (17.9) | 1.23 | 0.52-2.87 |  | 0.92 | 0.34-2.52 |
| Slept under insecticide-treated net the preceding night |  |  |  |  |  |  |  |
| No/unknown | 117 | 15 (12.8) | 1.00 |  |  | 1.00 |  |
| Yes | 541 | 86 (15.9) | 1.29 | 0.71-2.32 |  | 0.78 | 0.23-2.65 |
| Number of people sharing the same family type net |  |  |  |  |  |  |  |
| 1-2 people | 185 | 26 (14.1) | 1.00 |  |  | 1.00 |  |
| 3-4 people | 339 | 46 (13.6) | 0.95 | 0.57-1.60 |  | 1.10 | 0.63-1.92 |
| >5 people | 120 | 27 (22.5) | 1.78 | 0.98-3.22 |  | **2.22^c^** | **1.15-4.27** |

^a^: Odds ratio

^b^: 95% confidence interval

^c^: Bold numbers indicate statistically significant

## Logistic regression analysis which examined the associations of variables with malaria infection status in March survey (continued).

|  | Total | Positive  (Prevalence) | Bivariate | |  | Multivariate | |
| --- | --- | --- | --- | --- | --- | --- | --- |
|  | n=658 | n=101 (%) | OR^a^ | 95% CI^b^ |  | OR^a^ | 95% CI^b^ |
| Educational attainment |  |  |  |  |  |  |  |
| No | 123 | 13 (10.6) | 1.00 |  |  | 1.00 |  |
| Primary | 438 | 70 (16.0) | 1.61 | 0.86-3.02 |  | 1.01 | 0.49-2.08 |
| Secondary or above | 97 | 18 (18.6) | 1.93 | 0.89-4.16 |  | 1.09 | 0.45-2.69 |
| Rice farming type |  |  |  |  |  |  |  |
| Paddy only | 263 | 29 (11.0) | 1.00 |  |  | 1.00 |  |
| Slash-and-burn only | 128 | 26 (20.3) | **2.06** ^c^ | **1.15-3.67** |  | **2.12** ^c^ | **1.03-4.35** |
| Paddy and slash-and-burn | 267 | 46 (17.2) | **1.68** ^c^ | **1.02-2.77** |  | 1.69 | 0.96-2.98 |
| Distance to farming hut |  |  |  |  |  |  |  |
| <3.0 km | 214 | 30 (14.0) | 1.00 |  |  | 1.00 |  |
| >3.0 km | 444 | 71 (16.0) | 1.17 | 0.74-1.85 |  | 1.07 | 0.63-1.83 |
| Presence of hung net in main residence |  |  |  |  |  |  |  |
| No | 105 | 13 (12.4) | 1.00 |  |  | 1.00 |  |
| Yes | 553 | 88 (15.9) | 1.34 | 0.72-2.50 |  | 1.41 | 0.39-5.15 |
| Presence of hung net in farming hut |  |  |  |  |  |  |  |
| No | 357 | 49 (13.7) | 1.00 |  |  | 1.00 |  |
| Yes | 301 | 52 (17.3) | 1.31 | 0.86-2.01 |  | 0.91 | 0.54-1.54 |
| Village |  |  |  |  |  |  |  |
| A | 280 | 42 (15.0) | 1.00 |  |  | 1.00 |  |
| B | 209 | 45 (21.5) | 1.56 | 0.98-2.48 |  | 1.28 | 0.69-2.34 |
| C | 169 | 14 (8.3) | **0.51** ^c^ | **0.27-0.97** |  | 0.65 | 0.29-1.45 |

^a^: Odds ratio

^b^: 95% confidence interval

^c^: Bold numbers indicate statistically significant
