# Supplementary material for: Is staying overnight in a farming hut a risk factor for malaria infection in a setting with insecticide-treated bed nets in rural Laos?
Source: Malar J. 2010 Dec 23;9:372. doi: 10.1186/1475-2875-9-372 (PMC3224235; doi:10.1186/1475-2875-9-372)
Supplement: Additional file 2 — Logistic regression analysis which examined the associations of variables with malaria infection status in August survey. [file 1475-2875-9-372-S2.DOC]

## Additional file 2

## Logistic regression analysis which examined the associations of variables with malaria infection status in August survey.

|  | Total | Positive (Prevalence) | Bivariate | |  | Multivariate | |
| --- | --- | --- | --- | --- | --- | --- | --- |
|  | n=638 | n=146 (%) | OR ^a^ | 95% CI ^b^ |  | OR ^a^ | 95% CI ^b^ |
| Age (years) |  |  |  |  |  |  |  |
| <5 | 114 | 23 (20.2) | 1.00 |  |  | 1.00 |  |
| 5-14 | 219 | 52 (23.7) | 1.23 | 0.71-2.14 |  | 1.29 | 0.72-2.31 |
| >15 | 305 | 71 (23.3) | 1.20 | 0.71-2.04 |  | 1.33 | 0.76-2.32 |
| Sex |  |  |  |  |  |  |  |
| Female | 344 | 73 (21.2) | 1.00 |  |  | 1.00 |  |
| Male | 294 | 73 (24.8) | 1.23 | 0.85-1.78 |  | 1.31 | 0.89-1.93 |
| Number of household members |  |  |  |  |  |  |  |
| <5 people | 68 | 15 (22.1) | 1.00 |  |  | 1.00 |  |
| 5-9 people | 298 | 58 (19.5) | 0.85 | 0.45-1.62 |  | 0.93 | 0.46-1.89 |
| >10 people | 272 | 73 (26.8) | 1.30 | 0.69-2.44 |  | 1.73 | 0.77-3.87 |
| Household assets |  |  |  |  |  |  |  |
| No | 216 | 52 (24.1) | 1.00 |  |  | 1.00 |  |
| Radio and/or bicycle | 233 | 58 (24.9) | 1.05 | 0.68-1.61 |  | 1.17 | 0.71-1.94 |
| Motorbike and/or car | 189 | 36 (19.0) | 0.74 | 0.46-1.20 |  | 0.65 | 0.36-1.15 |
| Frequency of overnight stays in hut in the two weeks prior to the survey |  |  |  |  |  |  |  |
| 0 day | 174 | 39 (22.4) | 1.00 |  |  | 1.00 |  |
| 1-4 days | 153 | 44 (28.8) | 1.40 | 0.85-2.30 |  | 1.66 | 0.90-3.05 |
| >5 days | 311 | 63 (20.3) | 0.88 | 0.56-1.38 |  | 0.75 | 0.43-1.32 |
| Slept under insecticide-treated net the preceding night |  |  |  |  |  |  |  |
| No/unknown | 33 | 5 (15.2) | 1.00 |  |  | 1.00 |  |
| Yes | 605 | 141 (23.3) | 1.70 | 0.65-4.49 |  | 0.72 | 0.22-2.46 |
| Number of people sharing the same family type net |  |  |  |  |  |  |  |
| 1-2 people | 227 | 48 (21.1) | 1.00 |  |  | 1.00 |  |
| 3-4 people | 309 | 72 (23.3) | 1.13 | 0.75-1.71 |  | 1.19 | 0.75-1.88 |
| >5 people | 96 | 26 (27.1) | 1.39 | 0.80-2.40 |  | 1.15 | 0.62-2.13 |

^a^: Odds ratio

^b^: 95% confidence interval

^c^: Bold numbers indicate statistically significant

## Logistic regression analysis which examined the associations of variables with malaria infection status in August survey (continued).

|  | Total | Positive (Prevalence) | Bivariate | |  | Multivariate | |
| --- | --- | --- | --- | --- | --- | --- | --- |
|  | n=638 | n=146 (%) | OR ^a^ | 95% CI ^b^ |  | OR ^a^ | 95% CI ^b^ |
| Educational attainment |  |  |  |  |  |  |  |
| No | 86 | 19 (22.1) | 1.00 |  |  | 1.00 |  |
| Primary | 446 | 100 (22.4) | 1.02 | 0.59-1.78 |  | 1.12 | 0.59-2.15 |
| Secondary or above | 106 | 27 (25.5) | 1.21 | 0.62-2.36 |  | 2.05 | 0.87-4.80 |
| Rice farming type |  |  |  |  |  |  |  |
| Paddy only | 267 | 50 (18.7) | 1.00 |  |  | 1.00 |  |
| Slash-and-burn only | 153 | 36 (23.5) | 1.34 | 0.82-2.17 |  | 1.33 | 0.69-2.55 |
| Paddy and slash-and-burn | 218 | 60 (27.5) | **1.65** ^c^ | **1.08-2.53** |  | 1.33 | 0.74-2.39 |
| Distance to farming hut |  |  |  |  |  |  |  |
| <3.0 km | 222 | 50 (22.5) | 1.00 |  |  | 1.00 |  |
| >3.0 km | 416 | 96 (23.1) | 1.03 | 0.70-1.52 |  | 0.65 | 0.39-1.07 |
| Presence of hung net in main residence |  |  |  |  |  |  |  |
| No | 98 | 20 (20.4) | 1.00 |  |  | 1.00 |  |
| Yes | 540 | 126 (23.3) | 1.19 | 0.70-2.02 |  | 0.89 | 0.48-1.68 |
| Presence of hung net in farming hut |  |  |  |  |  |  |  |
| No | 124 | 24 (19.4) | 1.00 |  |  | 1.00 |  |
| Yes | 514 | 122 (23.7) | 1.69 | 1.01-2.84 |  | 1.82 | 0.92-3.58 |
| Village |  |  |  |  |  |  |  |
| A | 272 | 49 (18.0) | 1.00 |  |  | 1.00 |  |
| B | 215 | 60 (27.9) | **1.76^c^** | **1.15-2.71** |  | 1.51 | 0.84-2.74 |
| C | 151 | 37 (24.5) | 1.48 | 0.91-2.39 |  | 0.87 | 0.42-1.80 |

^a^: Odds ratio

^b^: 95% confidence interval

^c^: Bold numbers indicate statistically significant
